# Rapid intravenous symptom-inhibiting fentanyl induction (SIFI) to optimize rotation onto oral opioid agonist therapy among individuals who use unregulated fentanyl: protocol for an open-label, single arm clinical trial

**DOI:** 10.1186/s13722-025-00586-7

**Published:** 2025-07-29

**Authors:** Pouya Azar, Martha J. Ignaszewski, Marianne Harris, Zoran Barazanci, Ruth Davison, James S. H. Wong, Anil Maharaj, Nickie Mathew, David Hall, Silvia A. Guillemi, Julie Foreman, Rolando Barrios, Julio S. G. Montaner

**Affiliations:** 1 Complex Pain and Addiction Services, Vancouver General Hospital, Vancouver, BC Canada; 2 Department of Psychiatry, University of British Columbia, Vancouver, BC Canada; 3 Department of Child and Adolescent Psychiatry, BC Children’s Hospital, Provincial Health Services Authority, Vancouver, BC Canada; 4 British Columbia Centre for Excellence in HIV/AIDS, Vancouver, BC Canada; 5 Department of Family Practice, Faculty of Medicine, University of British Columbia, Vancouver, BC Canada; 6 Pharmacokinetics Modeling and Simulation Laboratory, Faculty of Pharmaceutical Sciences, University of British Columbia, Vancouver, BC Canada; 7 BC Mental Health & Substance Use Services, Provincial Health Services Authority, Vancouver, BC Canada; 8 School of Population and Public Health, Faculty of Medicine, University of British Columbia, Vancouver, BC Canada; 9 Department of Medicine, Division of Infectious Diseases, Faculty of Medicine, University of British Columbia, Vancouver, BC Canada

**Keywords:** Fentanyl, Methadone, Opioid use disorder, Opioid substitution treatment

## Abstract

**Background:**

Most opioid use disorder (OUD) treatment guidelines target community medical settings, and the subsequent recommendations were established to prioritize safety and reduce diversion prior to the fentanyl era. For people with OUD who use unregulated fentanyl, slow induction onto opioid agonist therapy (OAT) with gradual dose titration is often ineffective or insufficient for reducing withdrawal symptoms and cravings, thereby hampering engagement and retention in treatment. Given the severe risks associated with continued use of the increasingly toxic unregulated drug supply, new and innovative approaches to the management of OUD are urgently needed. We have developed an alternative induction protocol, using a rapid intravenous symptom-inhibiting fentanyl induction (SIFI) to optimize rotation onto oral OAT.

**Methods:**

An open-label, single arm, prospective pilot clinical trial is being conducted in an outpatient setting to assess the safety, feasibility, and efficacy of a rapid symptom-inhibiting intravenous fentanyl induction protocol to establish starting doses of methadone or sustained-release oral morphine (SROM) based on individual opioid requirements, as a treatment strategy for individuals with OUD who use unregulated fentanyl. The primary outcome is safety, as defined by occurrence of study drug-related adverse events (including but not limited to opioid toxicity and QT interval prolongation) that require intervention during induction and the first 7 days on OAT. Secondary objectives are to determine whether the SIFI protocol will result in use of higher-than-standard starting doses of methadone and SROM, and to determine whether implementation of this protocol will be acceptable to participants and will result in reduced withdrawal symptoms, improved retention, and better long-term outcomes on OAT.

**Discussion:**

This is the first study to rapidly and objectively estimate opioid tolerance and use it to calculate individualized starting doses of oral OAT in an outpatient setting among people who use unregulated fentanyl. We predict that starting methadone or SROM with individually-tailored doses will lead to therapeutic target concentrations being achieved quickly, safely, and with good patient satisfaction. This approach has the potential to more effectively and safely initiate OAT, to minimize opioid withdrawal and cravings, and in turn to decrease unregulated fentanyl use and increase retention on life-saving OAT.

**Trial registration:**

ClinicalTrials.gov, NCT05905367; date of registration: June 15, 2023; latest update posted July 18, 2024. https://clinicaltrials.gov/study/NCT05905367

*Protocol version*: 4.0, April 22, 2024.

**Supplementary Information:**

The online version contains supplementary material available at 10.1186/s13722-025-00586-7.

## Background

Canada is facing an escalating epidemic of unregulated drug overdoses, with fatalities in unprecedented numbers. More than 42,000 opioid toxicity deaths occurred in Canada between January 2016 and September 2023 [1]. The province of British Columbia (BC) is the most severely affected, with an annual rate of 45.3 opioid toxicity deaths per 100,000 population compared with the Canadian average of 19.4 per 100,000 population in 2022 [1].

The major driver of the ongoing crisis of drug overdoses and deaths is the increasing toxicity of the unregulated drug supply, fueled by fentanyl and its analogues. Fentanyl, a potent opioid agonist, was developed in the 1950 s to fill a need for strong and rapid analgesia. Because of these characteristics, fentanyl is commonly used to treat chronic cancer pain or in anesthesia [2]. Initially present as an adulterant in unregulated drugs, especially heroin, fentanyl is now the substance of choice for many people with opioid use disorder (OUD) [3].

The harms associated with fentanyl use are related to its high potency and short duration of action, leading to very frequent use, severe cravings, and more difficult to manage withdrawal symptoms [2, 4, 5]. Fentanyl was involved in 82% of Canadian opioid toxicity deaths in 2023 [1]. In BC, fentanyl was detected (alone or in combination with other drugs) in 86% of unregulated drug toxicity deaths in 2021, a striking increase from 5% in 2012 [6].

Opioid agonist therapy (OAT) has been shown to be safe and effective for the treatment of OUD. When prescribed in therapeutic doses, OAT decreases morbidity and mortality due to overdose and other OUD-related harms including serious acute and chronic infections including human immunodeficiency virus (HIV) and hepatitis C [7]. However, the shift to fentanyl in the unregulated drug supply has significantly complicated the management of OUD. People who use fentanyl experience severe withdrawal symptoms, often compromising OAT initiation, retention and adherence [4, 8].

Effective OAT doses are highly variable, depending on a number of factors, including the individual’s opioid tolerance and interindividual variability in pharmacokinetics [9]. Unfortunately, assessment of opioid tolerance is challenging, and is typically subjective, relying on the patient’s self-report of the amount and frequency of their opioid use, and is further complicated by the unpredictability of the unregulated drug supply [4]. Unknown opioid tolerance increases the risk of opioid overdose with OAT, and sequential administration of methadone without reaching steady state may cause “dose stacking” and the potential for dose accumulation [10]. In view of these risks, current North American OUD treatment guidelines (developed when heroin was the dominant opioid in the unregulated market) take a cautious approach, recommending low starting doses and slow, gradual dose increases [7, 11]. The British Columbia Centre on Substance Use (BCCSU) and Ontario’s Mentoring, Education, and Clinical Tools for Addiction: Partners in Health Integration (META:PHI) initiative have updated their guidance to reflect the changing unregulated drug market, providing recommendations for more rapid, albeit still conservative methadone initiation and titration [10, 12].

However, as previously noted, fentanyl is highly potent and is being used in large doses. As a result, currently recommended oral OAT induction protocols provide an inadequate speed of OAT titration for a large proportion of individuals with OUD who use unregulated fentanyl. Prescribing OAT according to current recommendations is frequently associated with persistence of severe and recurrent cravings and withdrawal symptoms over the weeks to months before therapeutic target levels of OAT can be reached, with ongoing unregulated substance use during the lengthy titration period [4, 8]. Indeed, a recent analysis of BC health administrative datasets including more than 55,000 people with OUD showed that, while OAT engagement and retention were protective against hospitalization and death, methadone initiation in compliance with current clinical guidelines was associated with increased risk of death or hospitalization within the first 6 months [13]. Even maximum recommended OAT doses are often inadequate to meet the opioid requirements of people who use fentanyl, leading to poor retention in OAT programs and continued use of unregulated drugs, with their attendant risks of overdose and death, even among those who remain on OAT [4, 13, 14].

In an attempt to address this issue in the context of managing hospitalized individuals who use fentanyl, we pioneered a novel alternative induction protocol using a rapid intravenous symptom-inhibiting fentanyl induction (SIFI) for objectively determining opioid tolerance using medically-administered intravenous (IV) fentanyl [3]. Implementation of the SIFI protocol in hospitalized patients with OUD has proven feasible, safe, and effective in meeting the patients’ opioid requirements, and thereby effectively managing opioid withdrawal and averting patient-initiated discharge from hospital against medical advice. Herein we propose to conduct the first evaluation of the safety, efficacy, and acceptability of the SIFI protocol in an outpatient setting.

To this end, we will implement and evaluate our rapid IV fentanyl induction protocol among patients who use fentanyl and who are receiving services at Hope to Health, a community primary care clinic located in the Downtown Eastside neighborhood of Vancouver, the epicenter of the unregulated drug use epidemic in Canada.

In brief, we will use each individual’s calculated opioid tolerance to guide their transition onto OAT with oral methadone or sustained-release oral morphine (SROM), with the choice of agent based on a shared decision-making process between the patient and their clinician. Methadone or SROM starting doses will be individualized based on an objective assessment of the patient’s opioid requirements (with defined upper dose limits in the interests of safety), thus achieving therapeutic target levels more quickly than with standard OAT dosing according to current OUD treatment guidelines. We anticipate that opioid withdrawal and cravings will thus be more effectively managed, leading in turn to improved acceptability and retention on OAT.

## Study design

### Overview of study design

To assess the safety, feasibility, and efficacy of a rapid symptom-inhibiting IV fentanyl induction (SIFI) protocol, we plan to conduct an open-label, single arm, prospective clinical trial with a planned sample size of 50. The primary goal of this pilot study is to identify unforeseen problems with the SIFI procedures that would impact participant safety. If such a problem exists with a prevalence of 5% (i.e. potential to affect 1 out of 20 participants), it will be identified with 92% confidence in a pilot study including 50 participants [15].

Results of this pilot study will be used to inform a subsequent larger and more comprehensive investigation of the SIFI protocol as a treatment strategy for individuals with OUD who intentionally use unregulated fentanyl.

### Study objectives

The primary objective is to assess the safety and feasibility of an outpatient SIFI protocol for determining individualized starting doses of oral OAT with methadone or SROM. Safety will be assessed in the first instance by a descriptive analysis of adverse events (e.g. sedation, respiratory depression, hypoxia, QT prolongation) requiring intervention during the induction/maintenance period (study days 1 through 7).

Secondary outcomes include starting OAT doses, participant satisfaction, and presence of withdrawal symptoms, OAT retention, overdose, hospitalization, and death at 1 and 3 months. Longer term impact of the strategy will be assessed at 6 and 12 months post-induction. Secondary outcomes will be reported using descriptive and summary statistics. Participants’ responses to the treatment satisfaction questions will be reported qualitatively, and used to refine the SIFI procedure for future investigation.

### Study setting

The opioid overdose crisis has disproportionately affected under-served communities, including those with low socioeconomic status and complex medical and mental health issues. This study will be conducted at the Hope to Health Research and Innovation Complex in Vancouver’s Downtown Eastside (DTES), a neighborhood of approximately 18,000 residents with high rates of poverty, homelessness, mental illness, and drug use [16]. Residents of the DTES are disproportionately impacted by the ongoing opioid overdose crisis: in 2023, the rate of overdose deaths per 100,000 population in the DTES was 560.9, which was ten times higher than in the rest of the city of Vancouver (56.7) or in the province of BC as a whole (46.5) [17].

Hope to Health is an integrated facility that includes a low-barrier interdisciplinary Primary Care Clinic which currently supports 2800 active clients, and a co-located Supervised Consumption Site. The Supervised Consumption Site handles approximately 1000 visits per month, of which 90% involve use of opioids with or without other substances. This is a nurse- and peer-led service offering safe spaces to use unregulated or prescribed substances, as well as distributing harm reduction supplies and offering nursing care and referrals to the Primary Care Clinic or other services, as needed. Both the Primary Care Clinic and the Supervised Consumption Site support a safer drug supply program that offers clients the possibility of replacing their unregulated drug supply with prescribed pharmaceutical grade alternatives, including hydromorphone tablets or fentanyl patches.

### Recruitment

Study participants will be recruited from the Hope to Health Primary Care Clinic and the adjacent Supervised Consumption Site. The planned sample size of 50 should be feasible based on the number of clients who are currently registered in the Hope to Health Primary Care Clinic who have a diagnosis of OUD and who do not have an active OAT prescription or who are receiving a suboptimal OAT dose (based on their continued use of unregulated fentanyl). In addition, the clinic continues to register new clients from the DTES population where OUD and fentanyl use are highly prevalent, including referrals from the adjoining Supervised Consumption Site where over 90% of visits involve use of opioids with or without other substances.

The clinic medical team will identify clients who are potential candidates for OAT, based on a diagnosis of OUD, ongoing active use of unregulated street drugs, and the client’s goal to decrease their opioid use. With the client’s approval, the clinic team member will introduce the client to the study coordinator, who will explain the study and seek their informed consent to participate. Written informed consent will be obtained from each prospective participant prior to their undergoing any study-related procedures. A research team member who is not directly involved in the client’s care will review and explain the study protocol and informed consent form with the prospective participant. It will be made clear that their participation is voluntary and that they are free to choose not to participate, or to withdraw from the study at any time, without affecting their access to clinic services or the quality care they receive at the Hope to Health Primary Care Clinic. The study procedures and reasonably foreseeable risks and potential benefits will be explained in detail. After the client has had adequate time to read the REB-approved consent form (or have it read and explained to them) and has had any questions answered to their satisfaction, if they agree to participate they will sign the consent form in a private office in the presence of the person who discussed the study with them.

After informed consent has been obtained and screening evaluations (Table 1) have been completed, research staff will review the inclusion/exclusion criteria and determine the potential participant’s eligibility for the study.

**Table 1:** Schedule of assessments during screening and IV fentanyl induction phase on Day 1

	Screening	Day 1 before start of induction	5 min after each IV fentanyl dose	5, 10, and 15 min after last IV fentanyl dose
Informed consent	x			
Medical history	x			
Medication review	x			
UDT	x			
Pregnancy test^a^	x	(x)^b^		
Height, weight		x		
Record time of last opioid use		x		
ECG		x		
POSS		x	x	x
COWS		x	x	
HR, BP, RR, SpO2		x	x	x
Participant-readiness threshold		x		
MSQ for OUD meds	x	(x)^b^		

*IV* intravenous, *UDT* urine drug test, *ECG* electrocardiogram, *POSS* Pasero opioid-induced sedation scale, *COWS* clinical opiate withdrawal scale, *HR* heart rate, *BP* blood pressure, *RR* respiratory rate, *SpO2* oxygen saturation, *MSQ* Medication Satisfaction Questionnaire, *OUD* opioid use disorder

^a^For individuals of child-bearing potential

^b^If screening and baseline not on same day

### Study population

#### Inclusion criteria

- Age 19 years or older
- OUD of any severity by DSM-5 Clinical Diagnostic criteria [18]
- Intentional use of unregulated fentanyl by any route (e.g. injection, inhalation) by participant self-report
- Urine drug test (UDT) positive for fentanyl at screening or within 7 days prior to date of screening visit, to confirm the presence of fentanyl
- Clinical indication to start OAT with methadone or SROM, or recently started OAT and receiving daily doses of methadone ≤ 150 mg or SROM ≤ 1300 mg (demonstrated to be subtherapeutic for the individual as they are continuing to use unregulated fentanyl)
- If taking prescribed opioids for safer supply, willing to discontinue them starting on study Day 1 and for at least the first 7 days of the study
- Willing and able to provide written informed consent for study participation

#### Exclusion criteria

- Individuals who are pregnant or breast-feeding
- Currently receiving prescribed fentanyl in any form, e.g. fentanyl patch
- Previous participation in this study (previous receipt of SIFI in an in-hospital clinical setting is not exclusionary)
- Current use of buprenorphine extended-release (Sublocade®) in any dose
- Use of buprenorphine-naloxone (Suboxone®) within the previous 3 days

Potential participants will not be excluded based on presence of substances other than fentanyl in the screening UDT, nor on the basis of alcohol use disorder or any other substance use disorder. The intent is to make the protocol applicable in a real-world setting where the majority of individuals who have OUD use multiple other substances in addition to opioids.

## Study treatment

### Induction phase

**Fig. 1:**
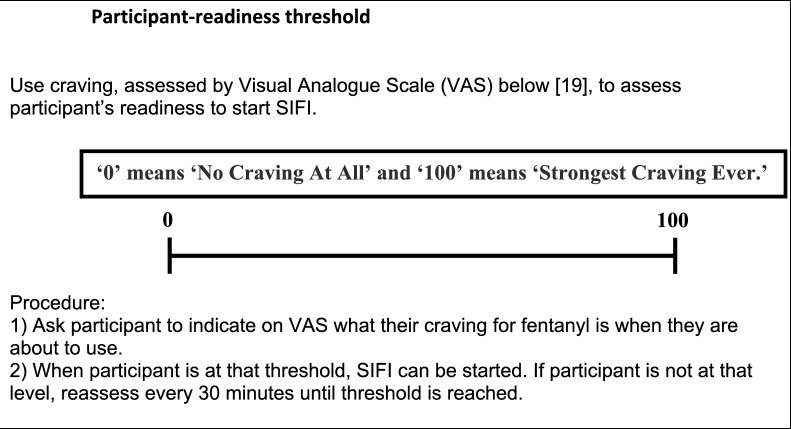
Participant-readiness threshold

Once the participant has provided informed consent and is found to be eligible based on results of the screening assessments (Table 1), the participant-readiness threshold will be used to determine the appropriate time to start the induction procedure according to the participant’s level of cravings (Fig. 1)[19].

When the participant reaches their readiness threshold, fentanyl 400 mcg will be administered intravenously (IV) by a study nurse or physician, with repeat doses every 5 min following assessment of sedation, withdrawal symptoms, and vital signs (Table 1) until the participant indicates comfort or has a Pasero Opioid-induced Sedation Scale (POSS) score of 2 (slightly drowsy, easily roused) [20]. After initial dosing with fentanyl 400 mcg per dose, stepwise increases to 800 mcg, 1000 mcg, and 1200 mcg per dose will be considered once tolerance and safety are established at each dose level. Sedation and vital signs will be reassessed at 5, 10, and 15 min after the final IV fentanyl dose, or until stable.

**Fig. 2:**
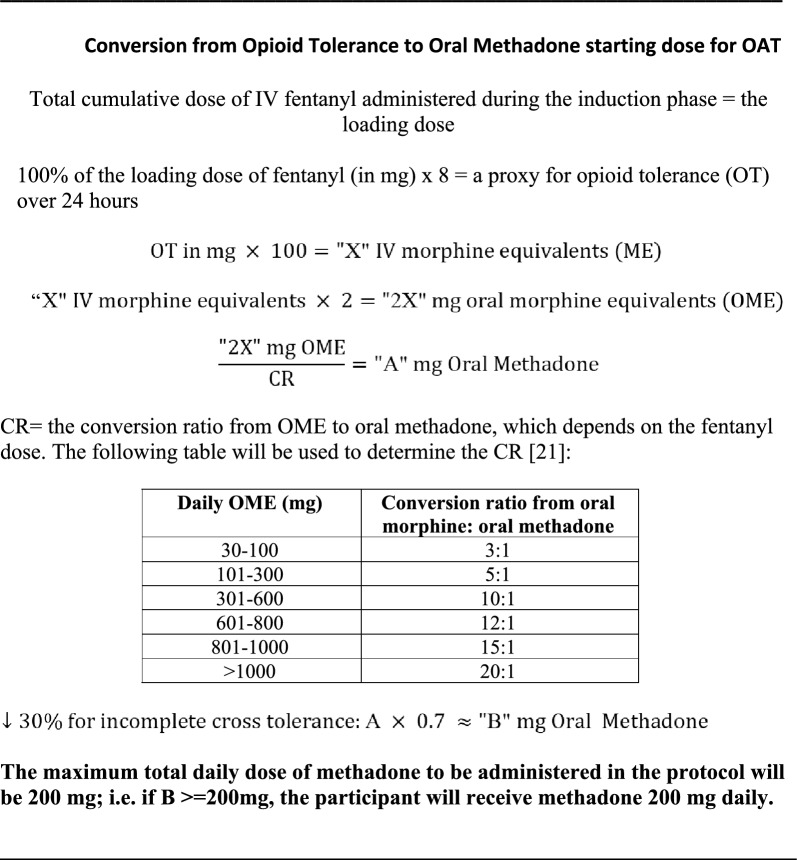
Conversion from Opioid Tolerance to Oral Methadone starting dose for OAT

**Fig. 3:**
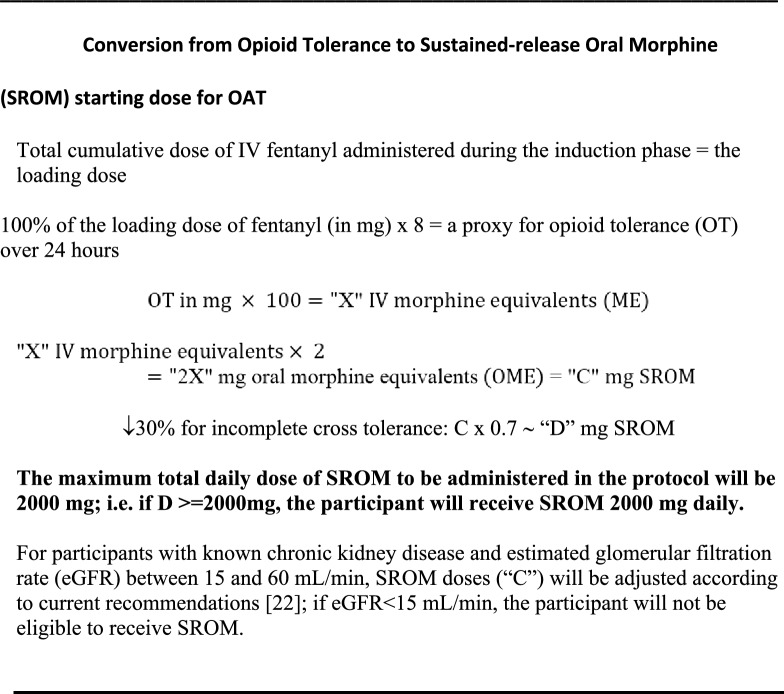
Conversion from Opioid Tolerance to Sustained-release Oral Morphine (SROM) starting dose for OAT

The total amount of fentanyl administered during the induction phase is the loading dose, to be used in the calculation of the individualized starting dose of methadone or SROM (Figs. 2 and 3)[21, 22].

The participant will remain under observation in the clinic while awaiting their first on-study dose of oral OAT.

### Early OAT phase

The specific OAT agent will be chosen prior to study enrolment by the clinic medical team in conjunction with the participant. To determine the first post-induction dose of OAT, the loading dose of fentanyl will be multiplied by 8 and used as a proxy for the participant’s opioid tolerance over 24 h. The multiplier of 8 in this calculation is based in part on anecdotal patterns of unregulated opioid use from the investigators’ experience—approximately 8 times per day. Since the protocol assessment of opioid tolerance reflects a single use, the multiplier of × 8 estimates total daily use. In addition, this approach aligns with the known duration of analgesia and respiratory depression from IV fentanyl boluses, which potentially last 1.5 to 4 h [23]. The calculated opioid tolerance will then be converted to the starting dose for methadone or SROM (Figs. 2 and 3), up to a maximum daily dose of 200 mg or 2000 mg, respectively. From the investigators’ clinical experience utilizing the SIFI protocol in an inpatient setting, participants are generally comfortable for approximately 1–3 h following induction [3], allowing adequate time for calculation, preparation, and dispensing of the individualized starting dose of OAT.

The first on-study oral OAT dose will be administered on the same day as the induction procedure and at least 15 min after the final IV fentanyl dose. On Day 1, participants will receive a maximum total methadone dose of 200 mg or a maximum total SROM dose of 2000 mg, adjusting for any previously prescribed methadone or SROM they received on Day 1 prior to arrival at the clinic. After the post-induction OAT dose is administered, the participant will remain in the clinic for 3 h under observation for ongoing monitoring of safety and vital signs (Table 2), and will receive a cash honorarium for their participation and for the time spent at the clinic. Thereafter, the participant will be offered honoraria for attendance at daily study visits for 7 days for assessment and OAT dispensing (Table 2). If the participant is found to be over sedated (POSS 3 or 4), reports sleeping more than usual, or exhibits any other signs or symptoms of excessive opioid levels, they will be referred to the clinical team for immediate assessment and management, including adjustment of OAT dose if deemed clinically necessary.

**Table 2:** Schedule of assessments during early OAT phase (Day 1 through Day 7)

	Before first OAT dose	1 h after first dose	2 h after first dose	3 h after first dose	Day 2	Day 3	Day 4	Day 5	Day 6	Day 7
HR, BP, RR, SpO2	x	x	x	x	x	x	x	x	x	x
POSS	x	x	x	x	x	x	x	x	x	x
COWS	x	x	x	x	x	x	x	x	x	x
Self-reported activity during previous 24 h					x	x	x	x	x	x
ECG	X^a^					X^b^				X^b^
MSQ for OUD meds	X^c^									x
MSQ + 3 questions re: SIFI				x						
Self-reported use of illicit opioids/other substances										x
UDT	X^c^									x
Overdose events req. intervention										x
Hospitalizations/death										x

*OAT* opioid agonist therapy, *UDT* urine drug test, *ECG* electrocardiogram, *POSS* Pasero opioid-induced sedation scale, *COWS* clinical opiate withdrawal scale, *HR* heart rate, *BP* blood pressure, *RR* respiratory rate, *SpO2* oxygen saturation, *MSQ* Medication Satisfaction Questionnaire, *SIFI* symptom-inhibiting fentanyl induction, *OUD* opioid use disorder

^a^For all participants, before start of IV fentanyl induction

^b^Only for participants receiving methadone

^c^At study screening

Methadone recipients who have QT prolongation at their follow-up ECG on Day 3 or Day 7 will be referred to the clinic team for assessment and management. For participants with a stable QTc and without evidence of opioid toxicity, methadone will be maintained at the same dose for the first 7 days.

For participants receiving SROM who meet all the following criteria, SROM doses may be increased by 100 mg every 24 to 48 h as clinically indicated, consistent with current local clinical guidelines [12].

- Presence of cravings and/or withdrawal symptoms
- No evidence of opioid toxicity (e.g. excessive sedation/lethargy, respiratory depression, bradycardia)
- No evidence of SROM adverse events (e.g. constipation, nausea, vomiting, dyspepsia, abdominal pain, urinary retention, headache, dizziness, hypotension, diaphoresis, xerostomia, dental pain, dysphoria, insomnia) [24]

The magnitude of the SROM dose increase will be based on clinical judgement, taking into account the starting dose and severity of ongoing cravings or withdrawal symptoms.

### OAT follow-up phase

After the first 7 days, OAT will be prescribed at intervals and doses adjusted as indicated according to the standard clinic protocols, and dispensed by a community pharmacy of the participant’s choosing.

Participants will be asked to refrain from using prescribed opioids for safer supply during the first 7 days of the study, but thereafter concomitant care and interventions will be provided as clinically indicated.

## Outcomes and assessments

### Timeline of assessments

Tables 1, 2, 3

**Table 3:** Schedule of assessments during OAT follow-up phase (months 1–12)

	1 month	3 months	6 months	12 months
Currently on oral OAT? (Yes/no)	x	x	x	x
Current OAT type and dose	x	x	x	x
Participant satisfaction (MSQ for OUD meds)	x	x	x	x
Self-reported use of illicit opioids/other substances	x	x	x	x
Clinic attendance	x	x	x	x
COWS	x	x	x	x
UDT	x	x	x	x
Overdose events req. intervention	x	x	x	x
Hospitalizations/death	x	x	x	x

*OAT* opioid agonist therapy, *UDT* urine drug test, *COWS* clinical opiate withdrawal scale, *MSQ* Medication Satisfaction Questionnaire, *OUD* opioid use disorder

### Primary outcome

The primary outcome of this study is safety, as defined by occurrence of study drug-related adverse events (including but not limited to sedation, respiratory depression, hypoxia, QT interval prolongation) requiring intervention during the induction and early OAT phases (study days 1 through 7).

The participant’s level of sedation (POSS) and vital signs (heart rate and oxygen saturation by pulse oximetry, blood pressure, and respiratory rate) will be monitored before starting induction, throughout the induction phase, at 5, 10, and 15 min (or until stable) after the final IV fentanyl dose, before the starting dose of OAT is administered, and hourly during the initial 3 h following oral OAT administration. In addition, sedation and vital signs will be assessed once daily for the first 7 days after the initial OAT dose, and during OAT follow-up at 1 month, 3 months, 6 months, and 12 months (Table 3).

In case of suspected opioid overdose (excessive sedation [POSS 3 or 4], respiratory rate below 8 breaths per minute, oxygen saturation below 92% or the participant’s baseline, and/or other signs or symptoms) at any time during the induction or OAT phases or during OAT follow-up, no further IV fentanyl doses will be administered and management will commence immediately according to the clinic overdose response protocol, consistent with established local and provincial guidelines [25].

Oral methadone can cause prolongation of the QTc (heart rate-corrected QT interval), which in turn is a risk factor for a rare but potentially fatal arrhythmia, Torsades de Pointes (TdP) [26]. An electrocardiogram (ECG) will be performed before commencing the induction and the results will be considered, along with other TdP risk factors, in the clinical decision-making process of selecting methadone or SROM as the appropriate OAT option for that individual [26, 27]. Participants who will receive methadone in this study will be advised during the informed consent process that the risk of arrhythmia is increased with higher doses of methadone. The first 7 days after starting methadone is the time of maximum risk of clinical adverse events, with a peak risk on Day 3 [28]; therefore, an ECG will be repeated on OAT Days 3 and 7 for participants receiving methadone. Any occurrence of QTc prolongation (> 500 ms, or ≥ 60 ms increase from baseline) and/or symptoms consistent with TdP will be managed promptly, consistent with recommendations in national OUD treatment guidelines [27].

### Secondary outcomes

The secondary outcomes are related to efficacy and acceptability, specifically:

- Starting doses of oral OAT (methadone or SROM)
- Participant satisfaction with the SIFI procedure
- Participant satisfaction with their current OUD treatment
- Proportion of participants who are retained on OAT after 1, 3, 6, and 12 months
- Withdrawal symptoms before and during induction phase, during early OAT phase, and during OAT follow-up
- Overdose, hospitalization, death at 1, 3, 6, and 12 months

Participant satisfaction with the SIFI procedure will be assessed during the immediate post-induction observation period, both quantitatively, using the single-item Medication Satisfaction Questionnaire [29, 30], and qualitatively, using three open-ended questions [31].

Participant satisfaction with their current OUD treatment (including safe supply, and recognizing that their current OUD treatment may be “none”) will be assessed before starting induction and during OAT follow-up visits using the single-item Medication Satisfaction Questionnaire [29, 30].

Withdrawal symptoms will be measured by the Clinical Opiate Withdrawal Scale (COWS) [32] before and during induction phase, during the early OAT phase, and during OAT follow-up.

Retention on OAT will be collected by participant self-report during the OAT follow-up visits and from the participant’s medical record. Any occurrence of overdose, hospitalization, and death during follow-up will be obtained from the participant’s medical record in the clinic electronic medical record (EMR) database.

## Treatment discontinuation and study discontinuation

Participants are free to withdraw from the study at any time without having to provide a reason for their decision. This will not affect their future medical care or other services that they receive at the clinic. If a participant decides to withdraw from the study, the research staff will not collect any further data from them, including data from the clinic EMR.

Discontinuation from study treatment may occur at the participant’s request or in the case of clinically significant adverse reactions and/or other safety reasons. If the participant experiences signs or symptoms consistent with opioid overdose during the induction procedure, the induction will be stopped and the clinic overdose response protocol will be implemented immediately. Ongoing management of the participant following an overdose event, including starting doses of OAT if appropriate, will be at the discretion of the clinic medical staff.

If a suspected opioid overdose occurs during the observation period after the first OAT dose or during a daily visit during the first week on OAT, the participant will be immediately referred to the clinic medical team for assessment and management, including adjustment of the OAT dose if deemed clinically necessary.

Participants receiving methadone who are found to have QTc prolongation (> 500 ms, or ≥ 60 ms increase from baseline) on their ECG on Day 3 or 7 and/or symptoms of possible TdP will be referred to the clinic medical team for prompt evaluation and management. Adjustment of methadone dose or transition to SROM may be offered as appropriate, based on clinical judgement and current Canadian OAT guidelines [27].

## Data analysis and management

The primary safety outcome will be assessed in the first instance by a descriptive analysis of adverse events requiring intervention during the induction and early OAT phases of the study. Secondary outcomes will be reported using descriptive and summary statistics. Participants’ responses to the treatment satisfaction questions will be summarized, and the results used to refine the SIFI procedure for future investigation.

Participants are assigned unique study codes that are not derived from or related to their personal information. Study-related documents, biological specimens, and ECG reports will be labelled only with the participant’s unique study code, and not with their name, initials, or other identifying information. All study documentation will be kept in a secure area in locked cabinets.

Files that contain personal identifying information are kept securely in locked offices in electronic form, and access to these files are password restricted to the principal investigator and a limited number of designated research team members. Identifying information collected on the questionnaires will be removed and stored separately from the questionnaire responses. Participant consents and contact information for follow-up will also be stored separately in a secure area in locked cabinets.

Electronic data will be stored on a secure server. For this study, clinical and sociodemographic information will be drawn from routinely collected clinic and administrative data in the clinic electronic medical record (EMR). Clinicians and research staff access the EMR via a roles-based, credentialed, secure portal to the EMR system.

Data in the clinic EMR database is under the custody of the British Columbia Centre for Excellence in HIV/AIDS (608-1081 Burrard Street, Vancouver, British Columbia, Canada) and its Director, Dr. Julio Montaner (a co-investigator on this study). The EMR database is stored on a secure server on the Centre’s network, protected by a defense-in-depth system including an industry-standard firewall prohibiting unauthorized entry. Strict measures are in place to protect the privacy of individuals in the database. The Centre also utilizes a suite of privacy and security policies to ensure the proper protection, management and integrity of all data. All data will be de-identified for study analyses to limit the potential for disclosure of identifying information.

## Monitoring

This pilot study will not have an independent Data and Safety Monitoring Board. Investigators will keep track of adverse events as they occur, and will revise the protocol and informed consent if an excess of adverse events is observed. Specifically, the trial will be put on hold (no new enrollments), revised, and resubmitted to the REB for approval before resuming, in any of the following circumstances:

1. Two serious adverse events that are possibly related to study participation and that occur during the participant’s first week of the study, as serious adverse events are unlikely to be related to the study drugs after methadone or SROM have reached steady-state levels.
2. Study drug-related adverse events requiring intervention (including but not limited to those meeting the criteria for serious adverse events) in 5 or more of the first 10 participants; 10 or more of the first 20 participants; 15 or more of the first 30 participants; or 20 or more of the first 40 participants.

## Current status of the study

The study protocol and informed consent form have received approval from the University of British Columbia/Providence Health Care Research Ethics Board (UBC/PHC REB Number H23-00111). The use of safe drug supply, opioid agonist therapy (OAT), and experimental approaches such as IV fentanyl for the treatment of severe opioid use disorder at the Hope to Health Research and Innovation Centre have been approved by Health Canada as part of its Substance Use and Addictions Program. Study recruitment is ongoing.

## Discussion

This is the first study to objectively estimate opioid tolerance and use it to determine individualized starting doses of oral OAT for use in an outpatient setting among people who use unregulated fentanyl. This study was initiated in response the inadequacy of current OAT dosing recommendations to address the increasing contamination of the unregulated drug supply with high-potency opioids, mainly fentanyl and its analogues, resulting in an ongoing crisis of opioid overdose and death in BC and elsewhere.

The SIFI procedure involves an IV induction with medical fentanyl, guided by pharmacological principles [33, 34], and has been implemented successfully in an in-hospital setting [3]. To optimize participant safety in this outpatient study, participants will be closely supervised in the clinic during and after the IV fentanyl induction procedure.

A limitation of the SIFI protocol is that sedation observed during the induction procedure may be due to other factors in addition to medically-administered IV fentanyl. In a setting where multi-substance use is common, other contributing factors may include prescription, non-prescription, or unregulated sedating substances (e.g. benzodiazepines, alcohol), and/or catecholamine depletion as a consequence of chronic use of methamphetamine or other stimulants. Operationally, any somnolence or lethargy observed during the induction procedure is attributed to the administered IV fentanyl. If additional sedating factors are present, the consequence would be to underestimate the participant’s opioid tolerance and administer too low a dose of methadone or SROM to fully meet their opioid requirements. This limitation is unavoidable in the interests of participant safety in a setting where the potential effects of concomitant sedating substances cannot be determined or quantified.

Another potentially confounding factor in the calculation of opioid tolerance could be the presence of buprenorphine, an opioid partial agonist that is sometimes prescribed for the treatment of OUD. While current use of either buprenorphine extended-release (Sublocade®) or buprenorphine-naloxone (Suboxone®) is exclusionary in our protocol, it should be noted that buprenorphine extended-release (Sublocade^®^) has a long terminal half-life and relevant plasma concentrations can be maintained for up to 5 months after the last administration [35, 36]. If the SIFI protocol were to be implemented in a setting where these agents are in regular use, it may be prudent to ensure a 5-month interval has passed since the last Sublocade^®^ dose or to ensure buprenorphine is absent during urine drug testing, to mitigate the confounding effects of residual buprenorphine on the assessment of opioid tolerance using IV fentanyl.

We expect that application of the SIFI protocol will result in higher-than-standard starting doses of oral OAT. Accordingly, in the interests of participant safety and to prevent early withdrawal after OAT initiation, every effort will be made to ensure the protocol-planned assessments are completed during the 3 h after the first dose of OAT and the first 7 days thereafter. Participants will be offered an honorarium for remaining in the clinic for a full day including participation in the induction and 3 h of assessment time following the first OAT dose. Honoraria will also be provided for participants returning to the study clinic for daily assessments during the first week on OAT.

We expect that the potential harms associated with IV fentanyl induction and high-dose methadone or SROM in this protocol will thus be minimized, especially when compared to the known harms associated with OAT dropout and continued use of the unregulated drug supply. We predict that tailored starting OAT doses will lead to therapeutic target levels being achieved more quickly, thereby more effectively managing opioid withdrawal and cravings, and in turn leading to increased retention on life-saving OAT and decreased reliance on the toxic street drug supply. We believe the findings of this study will be generalizable to other clinical settings, particularly in jurisdictions where fentanyl use is widespread.

## Supplementary Information

Additional file 1

## Data Availability

After the study is finished, results will be published in medical journals and reports, and presented at scientific meetings and conferences. These materials will be made available on the British Columbia Centre for Excellence website at www.bccfe.ca. Results will only be reported as aggregate data. No information which could identify any individual study participant will be included in any such presentations or publications. The final study dataset will be made available on specific written request to the Principal Investigator. The required study data will be de-identified and only the unique study code will be used for individual participant data.

## Abbreviations

BC: British Columbia
COWS: Clinical Opiate Withdrawal Scale
ECG: Electrocardiogram
EMR: Electronic medical record
IV: Intravenous
MSQ: Medication Satisfaction Questionnaire
OAT: Opioid agonist therapy
OT: Opioid tolerance
OUD: Opioid use disorder
POSS: Pasero Opioid-induced Sedation Scale
SIFI: Symptom-inhibiting fentanyl induction
SROM: Sustained-release oral morphine
TdP: Torsades de Pointes
UDT: Urine drug test
