# Supplementary material for: Rapid intravenous symptom-inhibiting fentanyl induction (SIFI) to optimize rotation onto oral opioid agonist therapy among individuals who use unregulated fentanyl: protocol for an open-label, single arm clinical trial
Source: Addict Sci Clin Pract. 2025 Jul 29;20:58. doi: 10.1186/s13722-025-00586-7 (PMC12306136; doi:10.1186/s13722-025-00586-7)
Supplement: Supplementary file 1 — Additional file 1 [file 13722_2025_586_MOESM1_ESM.docx]

**Participant Information and Consent Form**

**Rapid IV symptom-inhibiting fentanyl induction (SIFI) to facilitate rotation onto oral opioid agonist therapy (OAT)**

| **Principal Investigator** | **Pouya Azar**, MD, FRCPC, DABAM |
| --- | --- |
|  | Department of Psychiatry, Faculty of Medicine, University of British Columbia, Vancouver General Hospital, and Providence Health Care |
|  | xxx-xxx-xxxx |
| **Study Coordinator** | Zoran Barazanci |
|  | British Columbia Centre for Excellence in HIV/AIDS |
|  | xxx-xxx-xxxx |

**Emergency Telephone Number**

Critical or life-threatening condition 911

Hope to Health Clinic switchboard (24 hours) xxx-xxx-xxxx

**Non-emergency Contact Number**

For questions related to the study, please contact the study coordinator xxx-xxx-xxxx

For questions related to ongoing treatment, please contact your treatment provider

**1. Invitation**

You are being invited to take part in this research study because you are a client of the Hope to Health primary care clinic and/or the Supervised Consumption Site (SCS), you use fentanyl, and you are seeking treatment for opioid use disorder (OUD).

**2. Your participation is voluntary**

Your participation is voluntary. You have the right to refuse to participate in this study. If you decide to participate, you may withdraw from the study at any time. If you choose not to participate or if you withdraw from the study, your decision will not affect the medical care, education, or other services to which you are entitled or are presently receiving.

You should be aware that there is a difference for both you and your doctor between being a patient and being a research participant. As a patient, all medical procedures and treatments are carried out for your benefit only according to standard accepted practice. As a research participant, some procedures and treatments may not be part of standard practice or are not yet proven. This consent form describes the diagnostic and treatment procedures that are being carried out for research purposes. Please review the consent document carefully when deciding whether or not you wish to be part of the research and sign this consent only if you accept being a research participant.

If you wish to participate in this study, you will be asked to sign this form. Please take time to read the following information carefully and to discuss it with your family, friends, and doctor before you decide.

**3. Who is conducting this study?**

This study is being conducted by the BC Centre for Excellence in HIV/AIDS and the Complex Pain and Addiction Services at the Vancouver General Hospital.

This study is receiving funding from Health Canada’s Substance Use and Addictions Program.

**4. Background**

The standard treatment for opioid use disorder in Canada is opioid agonist therapy (OAT). The OAT agents used most often are methadone liquid or slow-release oral morphine capsules (SROM, brand name Kadian). Usually, OAT is started in low doses and the doses are increased slowly until the patient no longer has cravings or withdrawal. However, people who use fentanyl have worse withdrawal than people who use older opioids like heroin. The standard OAT doses may not be enough for people who use fentanyl, and they will often keep having cravings and withdrawal for several days or weeks until their dose of OAT is high enough to control their symptoms. Because of this, people who use fentanyl will often give up on OAT and go back to using unregulated/street drugs.

We are testing a way to determine the amount of methadone or SROM needed to control pain, withdrawal, and cravings for people who use fentanyl. This will be done by giving measured amounts of fentanyl through a vein (IV) every 5 minutes until the patient is comfortable and free of withdrawal symptoms. This procedure usually takes about 30 minutes and is called “symptom-inhibiting fentanyl induction” or SIFI. The amount of fentanyl given during the induction period is then used to calculate how much methadone or SROM they will need over a 24-hour period. This way we can tailor the starting doses of OAT to each person’s opioid needs.

We think this will lead to getting on the correct dose of OAT more quickly and being able to stay on OAT for longer than using the standard OAT dosing. However, it is not clear if SIFI is safe when used outside of hospital, and if it can offer better results than standard OAT dosing.

**5. What is the purpose of the study?**

The purpose of this study is to see whether giving IV fentanyl over a short period of time in clinic using the SIFI protocol, and using it to determine starting doses of OAT (methadone or SROM), is safe for people who use fentanyl. Another purpose is to see if SIFI will lead to better results in terms of staying on OAT and decreasing street drug use and overdoses over the next 12 months, in comparison to standard OAT dosing.

Health Canada has approved the sale or use of IV fentanyl to treat pain; although they have not approved its use for the management of opioid use disorder, they have allowed its use in this clinical study.

The goal is to enroll 50 participants in the study.

**6. Who can participate in this study?**

You may be able to participate in this study if*:*

- You are 19 years of age or older
- You are seeking treatment for opioid use disorder (OUD)
- You use street drugs including fentanyl by any route (injection and/or inhalation)
- Your urine drug test is positive for fentanyl at the screening visit or within the previous 7 days
- You are willing to stop taking any prescribed safer supply opioids (e.g. hydromorphone) you are currently taking, starting on study Day 1 and until at least study Day 7
- You provide written informed consent to participate in this study

**7. Who should not participate in this study?**

You will not be eligible to participate in this study if:

- You are pregnant or breast-feeding.
- You are currently receiving prescribed fentanyl in any form, e.g. fentanyl patch.
- You have previously participated in this study.
- You are currently taking more than 150 mg per day of methadone, more than 1300 mg per day of SROM, or any dose of buprenorphine extended-release (Sublocade®), or have taken buprenorphine-naloxone (Suboxone®) in the previous 3 days

**8. What does the study involve?**

**Screening visit (30-60 minutes)**

After you have signed this consent form, the study staff will find out if you are eligible to take part. To do this, they will ask you about the following:

- your use of street drugs, including which drugs you use and for how long, how often and how much.
- past overdoses and about your goals for treatment.
- your medical history, medications, and any medication allergies.
- if you have a history of chronic kidney disease, your kidney function will need to be checked with a blood test if you are going to take SROM, because the SROM dose may need to be reduced. If you have chronic kidney disease and are going to take methadone, no blood test or dose change will be needed.

Also during this visit:

- You will be asked to provide a urine sample for a drug screen, if this has not already been done in the previous 7 days. If you are able to become pregnant, the same urine sample will be used to do a pregnancy test.
- You will be asked to rate your satisfaction with your current treatment for opioid use disorder, on a scale from 1(extremely dissatisfied) to 7 (extremely satisfied).

Study staff may also get some of this information from your medical chart in the Hope to Health Clinic electronic medical record (EMR).

The screening procedures are part of standard medical care for people with OUD, except for the pregnancy test and the treatment satisfaction question. The informed consent discussion and screening visit will take about 30-60 minutes to complete.

**Baseline visit – Induction (SIFI) Phase (45-60 minutes)**

If you have signed the consent form are found to be eligible to participate in the study, the baseline visit can be done on the same day as the screening visit or can be scheduled for another day. The assessments and procedures done at the baseline visit are being done for study purposes only and are not part of standard medical care.

Before the SIFI procedure is started, the following will be done by the study staff:

- A urine pregnancy test will be done for people who are able to become pregnant, if this is not the same day as the screening visit or if one was not done at the screening visit
- Your height and weight will be measured
- You will be asked what time you last used fentanyl
- An electrocardiogram (ECG) will be done to check your heart, unless you already had an ECG done in the clinic within the last 7 days. An ECG is a painless procedure which records your heart rate and rhythm, and takes about 10 minutes to complete.
- An IV catheter (a small plastic tube) will be inserted into one of your arm veins
- A painless device called a pulse oximeter will be attached to your finger for the duration of the procedure, to measure your heart rate and the amount of oxygen in your blood
- Study staff will assess your level of sedation (drowsiness), withdrawal symptoms, breathing rate, and blood pressure

You will be asked what your craving for fentanyl is like when you are about to use, on a scale from 0 (no craving at all) to 100 (strongest craving ever), and to use the same scale to grade your current level of cravings. If your current level of cravings is below the point when you usually use, it will be rechecked every 30 minutes. When your cravings reach the point where you usually use, the SIFI procedure will start, as described below.

- A measured amount of fentanyl will be given into the IV catheter in your arm by a study nurse or doctor every 5 minutes, until you tell them you are comfortable or you become drowsy.
- After each dose, study staff will check your sedation level, withdrawal symptoms, blood pressure, breathing rate, heart rate and the amount of oxygen in your blood (from the pulse oximeter).
- If you become overly drowsy, your breathing is too slow, or the amount of oxygen in your blood is too low, no more fentanyl doses will be given and the Hope to Health clinic staff will take steps needed to prevent an overdose. This is a standard procedure in the clinic and may require giving you naloxone (Narcan).
- After the last induction dose, study staff will check if you have any withdrawal symptoms, and the IV catheter will be removed.
- Study staff will check your sedation level, withdrawal symptoms, blood pressure, breathing rate, heart rate and the amount of oxygen in your blood 5 minutes after the last dose of IV fentanyl. They will continue to monitor your heart rate and oxygen level for the next 15 minutes, then if these remain stable the pulse oximeter will be removed.

**Baseline visit - OAT Starting Phase (3 hours)**

- The choice of whether it is better for you to use methadone or SROM will have been decided before by you and the Hope to Health medical team. Prescribed safer supply opioids (e.g., hydromorphone) will be stopped at this time.
- The procedures described below for the OAT starting phase are being done for study purposes only and are not part of standard care for people starting OAT.
- After the study staff check your sedation level, withdrawal symptoms, blood pressure, breathing rate, heart rate and the amount of oxygen in your blood, you will be given your first dose of OAT to take in the clinic, and study staff will record the time you take it. You will be asked to stay in the clinic for 3 hours after your first dose. This is important to make sure that you are safe after starting SROM or methadone at doses that are higher than the standard OAT doses.
- Study staff will take you to a relaxation room in the clinic while you are waiting, and will check you about once an hour for your sedation level, withdrawal symptoms, blood pressure, breathing rate, heart rate and the amount of oxygen in your blood.
- If you become overly drowsy or have any other signs of opioid levels being too high, you will be seen by the Hope to Health clinic team who will take steps needed to prevent an overdose. This is a standard procedure in the clinic and may require giving you naloxone (Narcan).
- During this observation period, study staff will ask you to rate your satisfaction with the SIFI procedure (on a scale from 1=extremely dissatisfied to 7=extremely satisfied). They will also ask you: What did you like about the induction process? What did you dislike about the induction process? What would you change about the induction process?

Once all the assessments are complete at the end of the 3-hour observation period, you can leave the clinic. All participants will be asked to return to the clinic once each day for the next 7 days to receive their daily OAT dose and to have some assessments, as described below.

**Follow-up Phase – first days after SIFI induction and first dose of OAT (7 daily visits, each 15-30 minutes)**

- You will be asked to return to the Hope to Health clinic to receive your OAT dose once daily for the first 7 days. Other than dispensing medications, the assessments during these daily visits are being done for study purposes only and are not part of standard medical care.
- At each of these visits you will be seen briefly to the study staff to see how you are doing and to check your sedation level, withdrawal symptoms, blood pressure, breathing rate, heart rate and the amount of oxygen in your blood (using a pulse oximeter). They will ask how you spent the 24 hours since getting your dose of methadone or SROM, to assess whether you have been sleeping more than usual.
- If you are overly drowsy, have been sleeping more than usual, or have any other signs of opioid levels being too high, you will be seen by the Hope to Health medical team who will take steps needed to prevent an overdose. This is a standard procedure in the clinic and may require giving you naloxone (Narcan).
- If you are doing well, you will receive the same dose of methadone or SROM each day for the first 7 days, unless you are having cravings or withdrawal symptoms. In this case the dose of methadone or SROM may be increased after consulting with the Hope to Health medical team.
- If you are taking methadone, an ECG will be done on Day 3 and Day 7. If either of these days falls on a weekend or holiday, the ECG can be done 1 or 2 days before or after Day 3 or Day 7.
- On Day 7, you will be asked to rate your satisfaction with your current treatment for opioid use disorder, on a scale from 1 (extremely dissatisfied) to 7 (extremely satisfied). You will be asked about your current use of street drugs, including which drugs you use and for how long, how often and how much. You will be asked to provide a urine sample for a drug screen. You will be asked if you have had any overdoses or been in hospital during the last 7 days. These assessments will be done on Day 8 or 9, if Day 7 falls on a weekend or holiday. However, you will still be asked to see the study staff on Day 7 to check on your sedation level, withdrawal symptoms, blood pressure, breathing rate, heart rate and oxygen level, and recent activity level, as on Days 2 through 6.
- After the first 7 days, your daily OAT dose will be dispensed at a community pharmacy.

**Follow-up Phase – 1, 3, 6 and 12 months after SIFI and starting OAT (4 visits, each 15-30 minutes)**

You will be asked to return to the clinic 1 month, 3 months, 6 months, and 12 months after the SIFI baseline visit. At each of these visits:

- you will be asked if you are currently taking OAT and/or prescribed safe supply, and if so, what kind and how much
- you will be asked to rate your satisfaction with your current treatment for opioid use disorder, on a scale from 1 (extremely dissatisfied) to 7 (extremely satisfied)
- you will be asked about your current use of street drugs (opioids and/or other substances), including which drugs you use and for how long, how often and how much
- study staff will assess you for any withdrawal symptoms
- you will be asked to provide a urine sample for a drug screen
- you will be asked if you have had any overdoses or been in hospital since your last study visit

Study staff may also get some of this information from your medical chart in the Hope to Health Clinic electronic medical record (EMR) database. The treatment satisfaction question is being asked for study purposes only, while the other assessments and procedures at the follow-up visits are part of standard medical care for people with OUD.

**9. Pharmacokinetic (PK)/storage sub-study**

Pharmacokinetics (or PK) refers to the movement of a drug into, through, and out of the body. If you agree to take part in the main study, you will be invited to participate in an optional sub-study where timed blood and saliva samples will be collected to investigate the levels of fentanyl, hydromorphone, and methadone or morphine in your blood. If you agree to participate in the pharmacokinetic (PK)/storage sub-study you will be asked to sign a separate informed consent form in addition to the informed consent for the main study. The separate consent form will provide more information about the sub-study. If you choose not to participate in the PK/storage sub-study, you can still participate in the main study.

**10. Open Access**

The results of this study may be published in academic journals and presented at conferences. If this occurs, we may be asked to make the data publicly available. Any data released to the public will be de-identified and will not contain any information that could be linked back to you. Individual responses will not be shared, and only group results will be made available. Once the information is released, you will not be able to withdraw your data from the study. We acknowledge that the public release of data may increase the risk to your privacy, but we will assure that we keep your data confidential to the best of our ability.

**10. What are your responsibilities?**

As a participant in this study, you are expected to behave in a respectful manner toward members of the study team and staff of the Hope to Health primary care clinic and SCS. You will be responsible to adhering to the study protocol and presenting at the study site for your scheduled study appointments.

You should let the study team know about any kind of medication you use during the study period prescribed for you by health care providers outside the Hope to Health clinic.

**11. What are the possible harms and discomforts?**

Opioids and OAT including fentanyl, methadone, and SROM can cause overdose, especially when given in high doses. Opioid overdose can be fatal if not treated. Participants will be closely monitored throughout the SIFI procedure, and will be closely observed during the first 3 hours after taking their first dose of OAT. Participants will be assessed on a daily basis during the first week while receiving higher-than-standard OAT doses. Any participant who has signs or symptoms of possible opioid overdose will be assessed and managed by Hope to Health staff who are experienced in identifying and responding to suspected opioid overdose.

Methadone can cause a problem with the conduction of electric signals in the heart (detected by an ECG), which if severe can lead to an abnormal heart rhythm (arrhythmia) that rarely can be fatal. Precautions to prevent this happening are part of standard medical practice in treating people with OUD. Since you may receive a higher-than-standard dose of methadone during your participation in this study, the risk of arrhythmia may be increased. Therefore, people taking methadone in this study will be asked to have an additional ECG during the first week (i.e. one more ECG than for standard medical practice). The risks of performing an ECG are possible itching or irritation of the skin where the machine patches are placed.

An IV catheter (soft plastic tube) will be inserted into a vein in your arm for administration of IV fentanyl during the SIFI induction procedure during the Baseline/ Day 1 visit, and removed immediately after the induction is complete (about 30-60 minutes). Risks associated with IV catheter insertion are rare (1%) and include pain, bruising, bleeding, infection, or discomfort at the insertion site. Precautions will be taken to minimize these risks.

For any side effects experienced throughout the study, you should inform the study staff immediately.

**12. What are the potential benefits of participating?**

No one knows whether or not you will benefit from this study. There may or may not be direct benefits to you from taking part in this study. However, if you decide to participate, you will be receiving treatment for opioid use disorder. The expected benefits are that the study procedure will assist participants in reducing fentanyl use. We hope that the information learned from this study can be used in the future to benefit other people with opioid use disorder who use fentanyl.

**13. What are the alternatives to the study treatment?**

If you choose not to participate in this study, there are other treatment options for OUD. You can discuss these options with your doctor and health care team before deciding whether or not to participate in this study. If OAT with either methadone or SROM is the best option for you, these can be started at doses and according to the current standards of care at the Hope to Health clinic, without undergoing the SIFI procedure.

**14. What if new information becomes available that may affect my decision to participate?**

If you choose to enter this study and at a later date a more effective treatment becomes available, it will be discussed with you. You will also be advised of any new information that becomes available that may affect your willingness to remain in this study. You may be invited to sign an amended consent to indicate your continued consent to participate in the study.

**15. What happens if I decide to withdraw my consent to participate?**

You may withdraw from this study at any time without giving reasons. If you choose to enter the study and then decide to withdraw at a later time, all information about you collected up to the point of your withdrawal (including, where applicable, information obtained from your urine samples) will be retained for analysis in order to protect the integrity of the research, which may benefit future research participants and patients. However, no further information will be collected.

**16. Can I be asked to leave the study?**

Violence against clinic staff and/or study team members will lead to your automatic withdrawal from the study. You may also be asked to leave the study if the study doctor judges it is not in your best interest to continue, or if you are unable to fulfill the requirements for the study, or for any other reason. If you are asked to leave the study, the reasons for this will be explained to you and you will have the opportunity to ask questions about this decision. The study doctor will arrange for you to continue your care outside of the study. The study may also be stopped at any time by the UBC Providence Health Care Research Ethics Board if new information rises about the safety of the study treatment. The reasons for study stoppage will be explained to you and you will have the opportunity to ask questions about this decision.

**17. Will my information be kept confidential?**

Your confidentiality will be respected. However, research records and health or other source records identifying you may be inspected in the presence of the Investigator or his or her designate by representatives of Health Canada and the UBC Providence Health Care Research Ethics Board for the purpose of monitoring the research. No information or records that disclose your identity will be published without your consent, nor will any information or records that disclose your identity be removed or released without your consent unless required by law.

You will be assigned a unique study number as a participant in this study. This number will not include any personal information that could identify you (e.g., it will not include your Personal Health Number, SIN, or your initials, etc.). Only this number will be used on any research-related information collected about you during the course of this study, so that your identity will be kept confidential. Information that contains your identity will remain only with the Principal Investigator and/or designate. The list that matches your name to the unique study number that is used on your research-related information will not be removed or released without your consent unless required by law.

Your rights to privacy are legally protected by federal and provincial laws that require safeguards to ensure that your privacy is respected. You also have the legal right of access to the information about you that has been provided to the sponsor and, if need be, an opportunity to correct any errors in this information. Further details about these laws are available on request to your study doctor.

In accordance with Canadian law, data from this trial will be kept for 15 years.

Studies involving humans now routinely collect information on race and ethnic origin as well as other characteristics of individuals because these characteristics may influence how people respond to different medications. Providing this information is optional.

Because this is a treatment study, your signed consent form will be included in your electronic medical record, and your healthcare team will be alerted that you are on a study. This is to ensure your healthcare team has a little information about the study so that they can treat you safely according to the study protocol.

Your family physician will be notified of your participation in the study so that your study doctor and your family doctor can provide proper medical care.

As described above (on page 4 of this document), while you are participating in this study, the study staff may need to access the clinic EMR to get medical information about you that is needed for this study. They may also use the EMR for other reasons directly related to the study, for example to check your attendance at clinic appointments and get your contact information if they need to reach you for study purposes (such as to remind you about study visits). The study staff may also enter information about your study participation into your medical record in the EMR. If you agree to participate in this study by signing this informed consent form, you are authorizing this access to the EMR by study staff.

A description of this clinical trial will be available on http://www.ClinicalTrials.gov. This website will not include information that can identify you. At most, the website will include a summary of the results. You can search this website at any time.

**18. What happens if something goes wrong?**

By signing this form, you do not give up any of your legal rights and you do not release the study doctor, participating institutions, or anyone else from their legal and professional duties. If you become ill or physically injured as a result of participation in this study, medical treatment will be provided at no additional cost to you. The costs of your medical treatment will be paid by your provincial medical plan.

In case of a serious medical event, please report to an emergency room and inform them that you are participating in a clinical study and to contact the study team (please see page 1).

**19. What will the study cost me?**

All research-related medical care and treatment and any related tests that you will receive during your participation in this study will be provided at no cost to you.

You will not be reimbursed for any expenses incurred, such as parking or transportation.

**20. Will I be paid to take part in this study?**

After the Baseline visit: Once all the assessments are complete at the end of the 3-hour observation period, you will receive $100 cash and can leave the clinic. You will not receive a partial payment if you leave the clinic before the required time period.

During the Follow-up phase (Days 1 through 7 and months 1, 3, 6, and 12): After all assessments have been completed at the end of each visit, you will receive $20 cash each day.

**21. Who do I contact if I have questions about the study during my participation?**

If you have any questions or desire further information about this study before or during participation, or if you experience any side effects, you can contact the Principal Investigator, Dr. Pouya Azar at xxx-xxx-xxxx..

**22. Who do I contact if I have any questions or concerns about my rights as a participant?**

If you have any concerns or complaints about your rights as a research participant and/or your experiences while participating in this study, contact the Research Participant Complaint Line in the University of British Columbia Office of Research Ethics by e-mail at xxxx@xxxxx or by phone at xxx-xxx-xxxx (Toll Free: 1-xxx-xxx-xxxx.) Please reference the study number (H23-00111) when calling so the Complaint Line staff can better assist you.

**23. After the study is finished**

If you are interested in receiving a copy of the study results, or for any reason want a copy of your individual responses, you may contact the Research Coordinator.

After your participation in the study is completed, you will still be eligible to receive standard-of-care treatment for opioid use disorder with methadone or SROM. However, if for any reason your methadone or SROM have been stopped and you need to restart treatment, you may not be able to receive the study treatment (SIFI) after your participation in the study is completed. There are several possible reasons for this, some of which are:

- The treatment may not turn out to be effective or safe.
- The treatment may not be approved for use in Canada.
- Your caregivers may not feel it is the best option for you.
- You may decide it is too expensive and insurance coverage may not be available.
- The treatment, even if approved in Canada, may not be available free of charge.

**Rapid IV symptom-inhibited fentanyl induction (SIFI) to facilitate rotation onto oral opioid agonist therapy (OAT)**

**Participant Consent**

My signature on this consent form means:

- I have read and understood the information in this consent form.
- I have had enough time to think about the information provided.
- I have been able to ask for advice if needed.
- I have been able to ask questions and have had satisfactory responses to my questions.
- I understand that all of the information collected will be kept confidential and that the results will only be used for scientific purposes.
- I understand that my participation in this study is voluntary.
- I understand that I am completely free at any time to refuse to participate or to withdraw from this study at any time, and that this will not change the quality of care that I receive.
- I understand that I am not waiving any of my legal rights as a result of signing this consent form.
- I understand that there is no guarantee that this study will provide any benefits to me.
- I authorize access to my health records as described in this consent form.

I will receive a signed and dated copy of this consent form for my own records.

I consent to participate in this study.

| Participant’s Signature | Name | Date |
| --- | --- | --- |
| ____________________ | ____________________ | ____________________ |

| a. Signature of Person Obtaining Consent  b. Study Role | Printed Name | Date |
| --- | --- | --- |
| a. ____________________  b. ____________________ | ____________________ | ____________________ |
